# When does sedentary behavior become sleep? A proposed framework for classifying activity during sleep-wake transitions

**DOI:** 10.1186/s12966-018-0712-2

**Published:** 2018-08-22

**Authors:** Bethany Barone Gibbs, Christopher E. Kline

**Affiliations:** Department of Health and Physical Activity, University of Pittsburgh, 32 Oak Hill Court, Pittsburgh, PA 15261 USA

**Keywords:** Sedentary behavior, Sleep, Activity monitoring

## Abstract

The Sedentary Behavior Research Network recently published a consensus definition for sedentary behavior as ‘any waking behavior characterized by an energy expenditure ≤1.5 metabolic equivalents, while in a sitting, reclining, or lying posture.’ While this is a great step toward theoretical and methodological unity, further clarity around issues of classifying sedentary behavior while in bed is needed, specifically during sleep-wake transitions. A thigh-worn inclinometer with a 24-h wear protocol is recommended for best practice assessment of sedentary behavior, but this method introduces challenges for activity classification and data reduction. The constant stream of data collection does not distinguish waking sedentary activities in bed, e.g., watching television or reading, from sleep. Moreover, correct classification during sleep-wake transitions is not well established. Sleep-related behaviors can include time spent trying to fall asleep (sleep onset latency), night awakenings while attempting to fall back asleep (wakefulness after sleep onset), and unsuccessful attempts to fall back asleep in the morning (wakefulness after sleep offset). While these behaviors technically fit into the current definition of sedentary behavior, sleep-related behaviors belong in the sleep domain, are a normal part of the sleep-wake cycle, and are not likely an intervention target for sedentary behavior reduction. For these reasons, we argue that sleep-related behaviors should not be classified as sedentary. The research implications of using this framework for classifying sedentary behavior via 24-h thigh inclinometers include that diaries must ask participants to report the time they got into bed, began attempting to fall asleep (‘lights out’), woke up for the day, and got out of bed for the day. Using these diaries, researchers must manually extract the relevant period of wakefulness (and remove sleep-related and sleep time). The importance of this more burdensome protocol for researchers and participants, and across various subject populations, should be evaluated in future research.

## Commentary

Sedentary behavior is an emerging risk factor for adverse health outcomes and premature mortality [1, 2]. Addressing a need for methodological unity, the IJBNPA recently published the Sedentary Behavior Research Network (SBRN)‘s Terminology Consensus Project which used an iterative approach with the large SBRN membership to develop definitions for concepts associated with sedentary behavior. Most notably, the project came to agreement on the only slightly modified definition of sedentary behavior as ‘any waking behavior characterized by an energy expenditure ≤1.5 metabolic equivalents, while in a sitting, reclining, or *lying* posture’ [3]. We applaud this work to seek consensus on definitions across the sedentary behavior research community. In this commentary, we intend to expand the discussion to transitions between sedentary behavior and sleep, propose to separate sleep-related behaviors from sedentary behavior, and discuss research implications for accurate monitoring of these distinct behaviors.

Settling on a definition for sedentary behavior provides direction for research assessment methodology. Thigh- worn inclinometers offer best practice sedentary behavior measurement that captures intensity and posture [4], with a recent review recommending 24-h wear protocols, over at least seven days, and using a participant diary for data cleaning [5]. Twenty-four hour protocols offer an advantage to daytime-only wear protocols, which may suffer from missed days of wear or, more seriously, biased periods of nonwear that may not reflect wear periods (e.g., long sedentary bouts in bed at night after monitor removal). Yet, using the recommended 24-h wear protocol generates a constant stream of data – including posture and movement during sleep. This results in a need to denote each point of transition between wakefulness and sleep, and vice versa. Various methods are currently used, ranging from using set times (e.g., 6 AM to 10 PM), using diary-reported times, or automated algorithms [5, 6]. To help inform the optimal procedure for identifying sleep-wake transitions during sedentary behavior quantification, a more in-depth consideration of sleep-to-wake and wake-to-sleep transitions during a 24-h monitoring period may be useful.

**Fig. 1:**
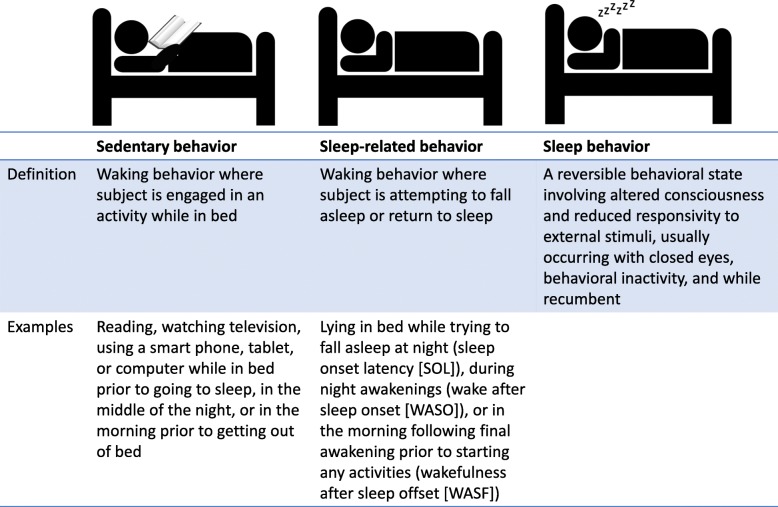
Clarification of in-bed behaviors: sedentary, sleep-related, and sleep

First, researchers should consider the variety of low-intensity behaviors that typically occur while awake in bed. Individuals may spend time in bed prior to sleep doing things other than attempting sleep, e.g., reading or watching television. These sedentary behaviors are not distinguishable from sleep in the output from the thigh-worn monitor, as only posture and movement are captured [4]. A further nuance is that, when in bed, individuals may spend time in what we propose to call ‘sleep-related’ behaviors. These can include trying to fall asleep after initial ‘lights off’ (sleep onset latency [SOL]), spending time awake in bed after initially falling asleep but prior to final morning awakening (wakefulness after sleep onset [WASO]), or lying in bed after final awakening trying to fall back asleep prior to getting up for the day (wake time after sleep offset [WASF]) (Fig. 1). The current SBRN definition of sedentary behavior [3] would technically include SOL, WASO, and WASF that occurred while lying in bed as a sedentary behavior since it meets the positional (lying down), intensity (very low), and contextual (awake) criteria of the definition.

Yet, sleep researchers might contend that these sleep-related behaviors are a normal, often healthy part of the sleep-wake cycle and should not be classified as sedentary behavior. While the overall physiology of these sleep-related behaviors may be similar to that of in-bed sedentary behaviors, the sleep-related behaviors belong in the sleep domain such that a basal amount of SOL (e.g., ≥ 5 to ≤15 min) is considered healthy and 30–60 min of WASO over a 7-h sleep period would be considered relatively normal [7, 8]. Moreover, adverse health effects associated with excessive SOL and WASO likely result through a pathway of insufficient duration or quality of sleep, rather than due to an accumulation of time spent in a low-intensity sitting, reclining, or lying posture [9]. A final, more practical argument is that SOL, WASO, and WASF occur with the intention of sleep and are not likely targets for interventions to reduce or break up prolonged sedentary behavior. It is likely that removing sleep-related behavior from sedentary time would have the biggest effect on sedentary time estimation in individuals with sleep disorders (e.g., insomnia) or poor sleep and may be trivial in healthy sleepers. Yet, because points of transition between sedentary behavior and sleep must be chosen when reducing 24-h thigh-worn inclinometer data, specifically to capture in-bed sedentary behavior, we argue that the ‘awake’ period should not include sleep-related behaviors as a rule.

Choosing these working definitions has implications for research practice in sedentary behavior measurement. Specifically, automated algorithms are likely to systematically miss in-bed sedentary behavior that is indistinguishable from sleep and sleep-related behaviors that occur during the same bout [6]. This in-bed sedentary behavior can be captured when diaries carefully (or explicitly) query participants for 1) the time they got into bed, 2) ‘lights out’ time (i.e., the time they began attempting to fall asleep), 3) the time they stopped attempting sleep, and 4) the time they got out of bed for the day. Yet, such diaries are burdensome for the participant and researcher during data reduction. The importance of capturing in-bed sedentary time in this way is not clear and may vary across study designs (e.g., epidemiological vs. within-subject measurement of sedentary behavior changes) and populations (e.g., adolescents vs. adults vs. older adults).

As sedentary behavior is increasingly considered and being measured in the context of 24-h behavior [3], future research should evaluate the importance of these more laborious methods to inform best practice. Modality and intention of in-bed sedentary behavior, for example reading a book for a soporific effect vs. several hours of television viewing, could also be important. Further, while the purpose of this commentary is to initiate discussion about whether sleep-related behaviors should be classified as sedentary behavior, future research should also consider whether to include upright awakenings and steps accumulated during the ‘sleep period’ (e.g., temporarily leaving the bed to go to the bathroom or due to difficulty sleeping) in daily totals. While the practice in our laboratory is to remove stepping and standing time that occurs during the ‘sleep period’ from daily totals, whether this is standard practice is less clear and should be systematically reported and evaluated.

In conclusion, we thank the SBRN for their diligent work toward a consensus on the definition of sedentary behavior and bring forth this further point of clarification (Table 1) in hopes of greater harmonization across sedentary behavior researchers. We look forward to other opinions and evaluation of the importance of specifically separating in-bed sedentary behavior from sleep-related and sleep behaviors for accurate sedentary behavior assessment.

**Table 1:** Key Points

1. Time spent in bed with low energy expenditure can be separated into three components: sedentary behavior, sleep-related behavior, and sleep. 2. Sleep-related behavior includes sleep onset latency (SOL), wakefulness after sleep onset (WASO), and wake time after sleep offset (WASF). 3. Though sleep-related behaviors would be currently classified as sedentary behavior (low energy expenditure, waking behavior in a lying position), we contend that sleep-related behaviors belong in the sleep domain; they are a normal part of a healthy sleep-wake cycle, are not a target for sedentary behavior reduction, and, when occurring in excess, likely increase health risk through distinct pathways (i.e., insufficient sleep duration or quality) from sedentary behavior. 4. For research using thigh-worn inclinometers and 24-h wear protocols, diaries that ask participants to report ‘time got into bed’ *and* ‘lights out’ as well as ‘time woke up and stopped attempting sleep for the final time’ *and* ‘time got up for the day’ are crucial for allowing researchers to separate sedentary time accumulated in bed from sleep-related behaviors and sleep.	

## Abbreviations

IJBNPA: International Journal of Behavioral Nutrition and Physical Activity
SBRN: Sedentary Behavior Research Network
SOL: Sleep onset latency
WASF: Wakefulness after sleep offset
WASO: Wakefulness after sleep onset
